# Dietary Implications of Polyunsaturated Fatty Acids during Pregnancy and in Neonates

**DOI:** 10.3390/life13081656

**Published:** 2023-07-29

**Authors:** Emilio Herrera, Henar Ortega-Senovilla

**Affiliations:** Facultad de Farmacia, Universidad San Pablo-CEU, CEU Universities, Urbanización Montepríncipe, Boadilla del Monte, 28660 Madrid, Spain

**Keywords:** pregnancy, fetal development, docosahexaenoic acid, arachidonic acid

## Abstract

Certain limitations exist for animals to modify fatty acid changes. Besides the role of arachidonic acid (AA), docosahexaenoic acid (DHA) and other 20-carbon long-chain polyunsaturated fatty acids (LCPUFAs) for the synthesis of inflammatory mediators as eicosanoids, different LCPUFAs have many other effects, including their abilities to regulate gene expression and downstream events. LCPUFAs are susceptible to autoxidation, which is prevented by the action of antioxidants in the form of enzymes like superoxide dismutases, catalases and peroxidases, as well as antioxidant compounds that protect against oxidation or repair the damage caused. Under normal conditions, the fetus needs both essential fatty acids (EFAs) and LCPUFAs, which are obtained from its mother by placental transfer. In early pregnancy, dietary derived fatty acids are accumulated in maternal adipose tissue. However, during late pregnancy, corresponding to the period of the highest fetal growth, maternal adipose tissue becomes catabolic and LCPUFAs are released into the circulation by adipose lipolytic activity. The released LCPUFAs are taken up by maternal liver to be esterified and released back to the circulation as triacylglycerides (TAGs) in very-low-density lipoprotein (VLDL) that become available to the placenta to be transferred to the fetus in the form of non-esterified fatty acids (NEFAs). An enhanced adipose tissue lipolysis is maintained around parturition and esterified LCPUFAs are diverted to mammary glands thanks to an increased activity of lipoprotein lipase for milk production. Throughout this process, LCPUFAs become available to the newborn during suckling. The important role of both DHA and AA for the development of the nervous system and for growth has motivated their dietary supplement during different postnatal stages. This has been especially important in preterm infants both because under normal conditions, the fetus acquires most of these fatty acids during late pregnancy, and because the immaturity of the enzyme systems for the synthesis of AA and DHA from their respective EFAs.

## 1. Introduction

Docosahexaenoic acid (DHA, 22:6 n-3) and arachidonic acid (AA, 20:4 n-6) are the two long-chain polyunsaturated fatty acids (LCPUFAs) known to play major roles in fetal development and pregnancy outcome [1]. These LCPUFAs are synthesized in different tissues from their respective two main precursor essential fatty acids (EFAs), α-linolenic acid (ALA, 18:3 n-3) and linoleic acid (LA, 18:2 n-6), that are present in the human diet. In the fetus, DHA and AA play an essential role in brain development, visual function and fetal growth [2,3], and together with eicosapentaenoic acid (EPA, 20:5 n-3), are the precursors of eicosanoids, including prostaglandins and leukotrienes, that can influence infant size [4,5]. This is the rationale for supplementing maternal diets with specific LCPUFAs or with fish-oils rich in omega 3 (n-3) LCPUFAs during pregnancy and lactation, recommending a total DHA intake of at least 300 mg/day for pregnant women and a daily supply of 200 mg of DHA during the lactation period by expert scientific organizations worldwide [6]. Thus, higher intakes of n-3 LCPUFAs [7] as well as a higher maternal n-3/n-6 LCPUFAs ratio [8] during pregnancy, have been associated with a higher duration of pregnancy and a reduction in the risk of premature birth, but also, with the fetal growth velocity from mid-pregnancy onward, better child neurodevelopment and an improvement in the overall health of the neonate [7]. These recommendations can be especially important in cases where the supply of omega 3 fatty acids could be compromised, such as in the case of vegetarian diets [9]. However, for a while, this practice was taken with caution, since some studies noted that a high intake of n-3 or n-6 dietary LCPUFAs may have adverse effects [10,11]: they may reduce antioxidant capacity as a consequence of an increased lipid peroxidation [12] and thereby cause an increased risk of oxidative damage, and so, a daily intake of LCPUFAs as >10% of total energy is not recommended [10]. The current studies do not support that reticence but show the benefit that n-3 consumption can have on inflammation and oxidative stress in pregnant women [13].

On the basis of the foregoing comments about the positive and potential negative effects of EFAs and LCPUFAs during the perinatal stages, this chapter provides a comprehensive overview of the metabolism of these fatty acids, their susceptibility to peroxidation and the implications for the offspring of including them in maternal diets during pregnancy and lactation.

## 2. General Aspects of the Metabolism of Polyunsaturated Fatty Acids (PUFAs)

### 2.1. Fatty Acid Chain Desaturation and Elongation

**Figure 1:**
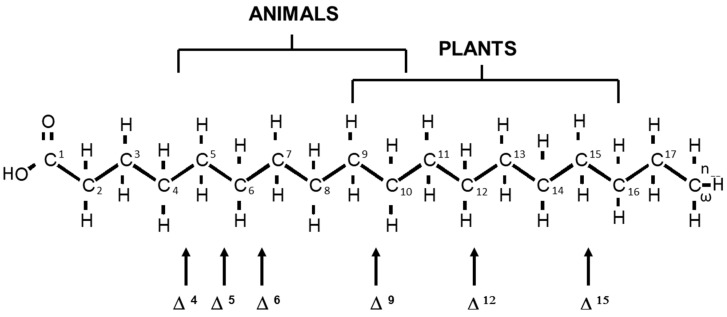
Positions of enzymatic fatty acyl chain desaturation in animals and plants. However, microscopic algae like *diatoms* and *Euglena* are able to desaturate on either side of an existing bond, yielding double bonds in positions Δ^5^, Δ^6^, Δ^9^, Δ^12^ and Δ^15^. Double bonds are indicated by the Δ, with the superscript indicating the first carbon number of the two affected carbons, counting from the carboxyl end of the molecule, or by n-3, n-6 and n-9 (also known as ω-3, etc.), counting from the terminal methyl carbon atom.

Animals can elongate and introduce double bonds into fatty acyl chains through well-known mechanisms [14,15], which differ between species and even between tissues. Although plants can introduce double bonds between an existing double bond and the terminal methyl group, animals can insert new double bonds only at the carboxyl end of the chain, and therefore cannot alter the n-family designation. Consequently, the n-family of the different fatty acids in our body cannot be modified endogenously and depends only on their dietary intake. Conjugated polyunsaturated fatty acids are those in which at least one pair of double bonds is separated by only one single bond. The introduction of conjugated double bonds is extremely rare in high animals, where most pairs of double bonds are separated by CH_2_ groups. Consequently, as shown in Figure 1, animals can insert double bonds at positions Δ^4^, Δ^5^, Δ^6^ and Δ^9^, whereas plants can insert at positions Δ^9^, Δ^12^ and Δ^15^. However, microscopic algae like *diatoms* and *Euglena*, are able to desaturate on either side of an existing bond, allowing the insertion of double bonds in positions Δ^5^, Δ^6^, Δ^9^, Δ^12^ and Δ^15^. Although several promising sources of EFAs have emerged [16], the consumption of oily fish is the main source of PUFAs in human nutrition and is commonly used for supplement production.

A desaturase system present in the endoplasmic reticulum of the cell is responsible for the fatty acyl chain desaturation, and although the best studied is the Δ^9^ desaturase, its mechanism of action is not yet completely understood.

Animals also elongate fatty acid chains by adding two carbon units at a time at the carboxyl end. Although mitochondrial elongation systems exist, the greatest elongation activity is in the microsomes of the endoplasmic reticulum, which contains at least one elongation system for saturated fatty acids and another for unsaturated fatty acids [17]. The substrates for fatty acid elongation are malonyl-CoA and a fatty acyl-CoA of 10 carbons or more.

**Figure 2:**
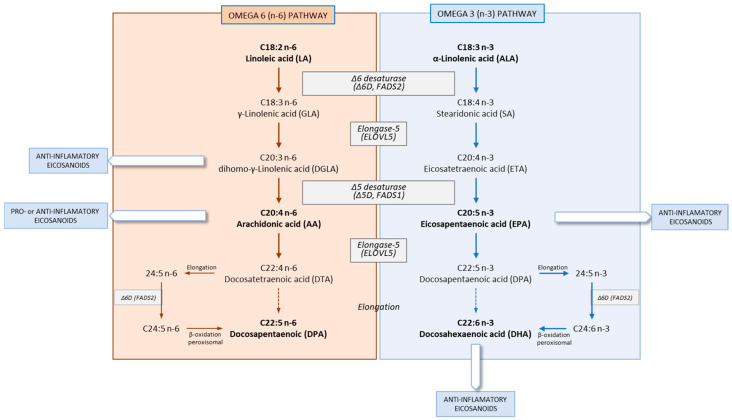
Major pathways in mammals of the biosynthesis of n-6 and n-3 long-chain polyunsaturated fatty acids (LCPUFAs) from their respective dietary-derived essential fatty acid precursors (EFAs) by desaturation and elongation, as well as the peroxisomal degradation reactions.

Under normal conditions, in mammals, there is an alternative activity of fatty acid elongation and desaturation that results in the synthesis of n-6 and n-3 LCPUFAs, from their 18-carbon unsaturated EFA precursors from the diet (Figure 2). This pathway requires the activity of desaturation and elongation reactions that are followed by partial β-oxidation degradation reactions that take place in peroxisomes [15,18]. The levels of n-6 LCPUFAs also have the ability to impact n-3 LCPUFAs status and vice versa. This is due to the competition between n-6 and n-3 LCPUFAs on the delta-6 desaturase (Δ6D), for the enzymatic conversion of both of the EFA precursors, LA and ALA, to their respective long-chain derivatives. Moreover, LA competes with ALA for Δ6D, and as a result, high levels of LA have the potential to reduce the efficiency of the endogenous synthesis of n3- LCPUFAs from ALA. Also, it has been suggested that increasing the dietary intake of ALA could provide an alternative to fish (or fish oil) intake for increasing n-3 LCPUFAs, although the efficiency of this process in humans is generally low [19]. In addition, it has been suggested that reducing LA and/or increasing ALA intake in humans, could increase EPA and, to some extent, DHA levels [20], although there is a need for well-controlled studies to corroborate this. Besides the diet, hormones [21] and age [22] modulate the desaturation and elongation processes.

The n-6 pathway synthesizes AA, from which the eicosanoids (prostanoids, leukotrienes and lipoxins) and longer-chain fatty acids are synthesized. The eicosanoids are physiologically and pharmacologically active compounds that are signaling molecules considered to be local hormones, which function through G-protein-linked receptors to produce their biochemical effects.

In the n-3 pathway, EPA and DHA are synthesized, which, together with AA, are esterified into the phospholipids of cell membranes, including those in the brain, where they have important clinical significance [23].

### 2.2. Association of Polyunsaturated Fatty Acids with Oxidative Stress

PUFAs of both n-6 and n-3 families have multiple unsaturated bonds and are highly susceptible to oxidation [5], which may have an impact on food quality and health. The process can be categorized into non-enzymatic oxidation and enzymatic oxidation.

**Figure 3:**
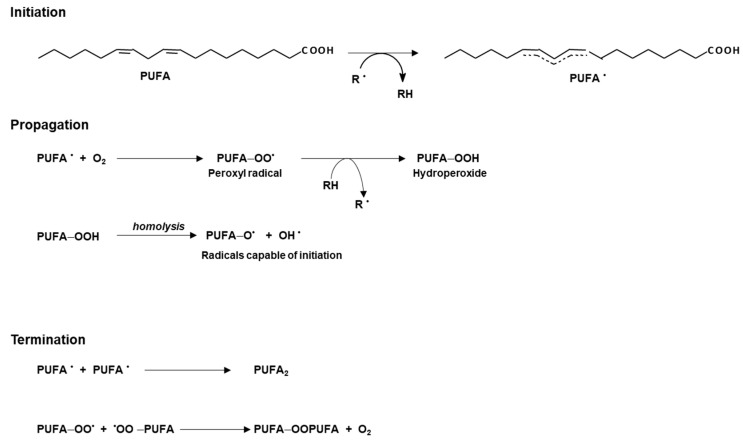
Autocatalytic oxidation process of a polyunsaturated fatty acid (PUFA). **R^•^**: Free radical; **PUFA^•^**: resonance hybrid; **PUFA-OO^•^**: peroxyl radical; **PUFA-OOH**: hydroperoxide; **PUFA-O^•^**: alkoxy radical; **OH^•^**: hydroxyl radical.

In the case of non-enzymatic oxidation, the process can be considered autoxidation, the reaction being mediated by free radicals, which may be formed under the influence of traces of peroxides, transition metals, ultraviolet or ionizing radiation and other factors. As summarized in Figure 3, a resonance hybrid is formed, which is already a free radical that may react with oxygen to form peroxy-radicals (PUFA-OO^•^), which may abstract a hydrogen atom from another substrate, converting the peroxy radicals to hydroperoxides (PUFA-OOH). Under certain conditions, the hydroperoxides decompose by homolysis, resulting in radicals that may initiate a new peroxidation chain. The termination of the process occurs when two hybrid radicals are coupled, forming a product that is no longer a free radical. The formation of both hydroperoxides and lipid peroxide degradation products may cause a disturbance to the structure of the cell membrane [24].

In enzymatic oxidation, the action of phospholipases (mainly phospholipase A2) cleaves phospholipids at the *sn*-2 position, releasing lysophospholipids and free PUFAs [25]. The released free PUFAs can be converted into prostaglandin H2 by the action of cyclooxygenases (COXs), also termed prostaglandin endoperoxide H synthases [26]. Prostaglandin H2 is unstable and can be converted to different prostanoids by the action of prostanoid synthases [26]. The free PUFAs can also be converted to hydroperoxides by the action of lipoxygenases (LOXs), which catalyze the insertion of molecular oxygen into PUFAs [27]. The products of the LOX reactions are potent lipid mediators. This includes their action on EPA that yield E-series resolvins (RvEs) and on DHA to form D-series resolvins (RvDs) [28]. The conversion of n-3 and n-6 PUFAs into the different series of prostaglandins, thromboxanes and leukotrienes is also catalyzed by COXs and LOXs. There are two major human cyclooxygenase isoforms, COX-1 and COX-2, although COX-2 needs lower concentrations of hydroperoxide for activation and has a wider substrate specificity, being responsible of those effects [5].

PUFA oxidation in the cell membrane may be even more complex [29]. The formation of lipid peroxide degradation products, like endoperoxides and malondialdehyde (MDA), may damage membrane structure. This can be particularly important in low oxygen environments, which favors the generation of molecules having one or more unpaired electrons, such as reactive oxygen species (ROS), enhancing oxidative damage. Early pregnancy is characterized by a low oxygen environment, where an excessive production of ROS might have taken place [30], and this has led to speculation that this process may be related to the development of certain gestational disorders. For example, in pregnant women with gestational diabetes, there are studies that describe that alterations attributable to oxidative stress (like higher serum MDA levels) are present before the biochemical detection of glycated hemoglobin (HbA1) [31]. The same has been observed in preeclampsia, where increments of serum MDA could be a useful biomarker to an early diagnosis of preeclampsia [32,33]. On the other hand, the fetal-to-neonatal transition is another situation of the risk of oxidative damage because the intermittent hypoxia periods involve the overproduction of ROS and promote lipid peroxidation [34]. Preterm neonates constitute a special case, as they are highly susceptible to oxidative stress due to an imbalance between the oxidant and antioxidant systems and increased levels of MDA as well as other final products of lipid peroxidation [35], which appear to be the main factors responsible for several diseases occurring in this population [36]. Initially, one might think that this situation could be aggravated with the use of lipid emulsion for the parenteral nutrition of preterm neonates, because studies in animal models have shown that in purified preparations, PUFAs in phospholipids are especially susceptible to peroxidation (albeit less so than free PUFAs) [37], noting an increase in MDA production and a decrease in vitamin E concentration after the intake of polyunsaturated oil [38,39]. However, actual lipid emulsion currently used in pediatric patients are safe and administered during short periods, do not produce significant changes on lipid peroxidation [40] nor increase MDA production [41]. Moreover, composite fish-oil-containing lipid emulsions, due to their high DHA and EPA content along with their high concentration of lipid antioxidant vitamin E, have the advantage of reducing oxidative stress/lipid peroxidation compared with either pure soybean or soybean/olive-oil-based emulsions, and also, have higher DHA and EPA levels [42,43]. Data regarding the effects of n-3 or n-6 PUFA supplementation on oxidative stress in pregnant women are limited. But based on the results of a recent meta-analysis [13], no significant effects of n-3 supplementation during pregnancy on MDA were found.

### 2.3. Antioxidant Defenses

During normal metabolism, other oxidant substances, like ROS, hydroxyl radicals and reactive nitrogen species, are also produced. These reactive molecules may directly affect proteins, lipids, nucleic acids and other compounds that can produce mutations, tissue damage, changes in enzyme activities, changes in signal transduction pathways and other effects that can cause clinical pathological conditions [44,45]. Moreover, there are certain situations such as perinatal resuscitation, mechanical ventilation and postnatal complications that, together with immature antioxidant capacities and the placental–fetal transfer interruption of antioxidant molecules, like α-tocopherol, may increase ROS production and contribute to the pathogenesis of several complications, where intraventricular hemorrhage, retinopathy and bronchopulmonary dysplasia (BDP) are recognized as organ-specific manifestations. In fact, it has been proposed that the early determination of lipid peroxidation in preterm infants could be a marker to predict some of postnatal free radical-associated conditions, such as BPD [46].

The continuous production of these highly reactive molecules can be compensated in the organism by antioxidant enzymes, through compounds that protect against the oxidation process, or through the repair of the damage caused. Within the antioxidative enzymes are the superoxide dismutases [47], catalases [48] and different peroxidases [49]. On most occasions, the action of these antioxidant enzymes is coordinated with the production of reactive species that are then destroyed.

The body also contains, at low concentrations, several antioxidant compounds that also protect against the generation of excessive aberrant radical species and their consequences. Some of these compounds are intracellular, but others are extracellular, mainly in plasma, where several antioxidant compounds, like albumin, α-tocopherol, carotene (α- and β-), reduced glutathione and others at different concentrations exist in normal and pathological processes. As mentioned above, pregnancy, labor and childbirth are associated with the excessive formation of free radicals, which can be scavenged by antioxidants in healthy pregnancy to prevent diseases or diminishing the effects of oxidative stress in pregnant women and neonates [50,51]. It is not the purpose of this review to show the differences that exist in antioxidant status between different tissues, or between mother and neonates. However, it is known that antioxidant concentrations are determined by many factors, including genetics, maternal diet, maternal body habitus and co-existent pathologies. However, the available components may be insufficient to respond to the increased production of free radicals, especially in tissues with high oxygenation activity during pregnancy, like placenta, promoting changes in the structure of cells and thus, causing damage with harmful effects on fetal and maternal cells. In fact, excessive ROS production is associated with several pregnancy complications, such as preeclampsia, fetal growth restriction, gestational diabetes and preterm birth [52], so in these populations, antioxidants could be a promising tool to counteract the inflammation and oxidative pathways [53]. Moreover, neonatal antioxidant status is lower than in the mothers’, with limited concentrations of glutation peroxidase, superoxide dismutase, vitamin E and other plasma factors, which may be inadequate to protect fetal tissues, especially in high-risk pregnancies and deliveries, including preeclampsia and the premature rupture of membrane. In those cases, the use of lipid emulsions in the parenteral nutrition of preterm neonates, enriched in α-tocopherol, help to reduce oxidative stress/lipid peroxidation [42]. Nevertheless, clinical evidence on their use, especially those of natural origin, is scarce and controversial [54], and the choice of the best antioxidant and the best dose to improve neonatal oxidative stress damage still remains unknown.

## 3. Principal Utilization of LCPUFAs by the Fetus

### 3.1. Importance of LCPUFAs for the Fetus

Although during the early stages of development—corresponding to the first 8 weeks of intrauterine life—PUFAs are required by the embryo, their rate of utilization is small and does not represent an additional demand on the mother or her diet. Indeed, until around 25 weeks of gestation, the accumulation of lipids and of specific fatty acids in the fetus is relatively small, but increases logarithmically with intrauterine age, reaching its maximal rate of accretion right before term [55].

During the last 5 weeks of gestion, the greatest increase in individual fatty acids occurs in the fetus. During this period, AA accumulates more rapidly in the fetus than DHA does, but a higher proportion of DHA than AA is found in cerebral tissue at term [3]. During pregnancy and the neonatal period, the supply of these fatty acids is critical and, in cases where the accumulation of DHA during the intrauterine period is insufficient, a permanent impairment of both retinal function and learning ability occurs.

A significant positive linear regression between birth weight and plasma AA (and total n-6 LCPUFAs) in premature infants has been found [56], and it is suggested that such an effect of AA is related to its structural roles in membrane phospholipids or it being the precursor of leukotrienes, prostaglandins and thromboxanes, collectively known as eicosanoids, that are important for immune response. It was also found that AA is critical for infant growth [57] and correlates with first-year growth in preterm infants [58].

### 3.2. Role of LCPUFAs in Neurological and Visual Development

LCPUFAs are essential for early brain development, being involved in both the structure and function of the nervous system. DHA and EPA are involved in brain functions, including neuronal membrane dynamics and neurotransmitter functions [23]. AA, besides other functions, protects the brain from oxidative stress [59] and is involved in early neurological development.

The greatest accretion of AA and DHA in the brain and retinal tissues occurs during the third trimester of intrauterine life [60]. Therefore, preterm delivery, which cuts short the supply of placental LCPUFAs, reduces their availability, consequentially compromising neurodevelopment. This is supported by reports on the selective transfer of those LCPUFAs by the placenta [61], the consumption of milk in breastfed infants [62] and their synthesis from the corresponding EFAs [63]. 

DHA has been shown to have a neuroprotective role during neonatal asphyxia in rats [64] and in prematurely born pigs [65]. In rats, a maternal diet enriched in DHA during pregnancy prevents neonatal brain injury [66]. In a study of “cot death” in human infants, it was found that in those fed exclusively breast milk, their DHA content in cerebral cortex phospholipids was greater than in those fed LCPUFAs-deficient formula, and that such deficiency in DHA was largely compensated for by an increased incorporation of n-6 fatty acids into neural tissues [67].

Besides abnormal peripheral neuropathy, an altered n-3 LCPUFAs pattern during late fetal and early postnatal life in humans has been also associated with abnormal visual function [68]. A decrease in severe retinopathy found in prematurity has been found in preterm infants receiving enteral DHA supplements [69].

Very-low-birth-weight (VLBW) neonates have practically no adipose tissue and consequently have decreased EFAs and LCPUFAs reserves. In fact, at day 5 of postnatal life, VLBW infants given fat-free diets show an EFA deficiency in plasma phospholipids [70]. Human neonates as young as 26 weeks of gestation can synthesize LCPUFAs from their precursors, although this conversion is quite limited and not sufficient to allow normal biochemical and functionality [71,72]. In fact, since the elongation–desaturation pathways seems to be low in early life, the dietary supply of EFAs in VLBW infants is not sufficient to satisfy the requirement of LCPUFAs for appropriate retinal function [68]. Moreover, a longitudinal study of VLBW children followed up to 20 years of age have shown that their low neurodevelopmental sequelae and low educational attainment persist until young adulthood [73].

### 3.3. Fatty Acids as a Source of Energy for the Fetus

It was considered for some time that the fetus depends primarily on glucose oxidation for energy production; however, more recently, it has been shown that both mRNA expression and activity of fatty acid oxidation (FAO) enzymes are high in both the placenta [74] and different human fetal tissues [75], indicating that FAO actively contributes to fetal and placental energy production. It has even been shown that disorders in genes of the FAO pathway are associated with newborn prematurity, intrauterine growth retardation, encephalopathy, coma and death [76]. These findings demonstrate that FAO is important in the human fetal–placental unit as a major producer of energy and has a critical role in fetal development.

Although all fatty acids can provide energy by mitochondrial oxidation, differences in the proportion of each fatty acid used by the fetus for oxidation exist. As previously discussed [77], LCPUFAs might appear to be the preferred oxidative substrate due to their more unsaturated condition. Moreover, since the primary LCPUFAs used by the fetus are of maternal origin compared to other fatty acids [55], it can be expected that LCPUFAs of maternal origin, which are the preferential substrate for placental transfer when compared to other fatty acids, are the preferred oxidative substrate for the fetus. 

## 4. Storage of Dietary LCPUFAs in Maternal Adipose Tissue

**Figure 4:**
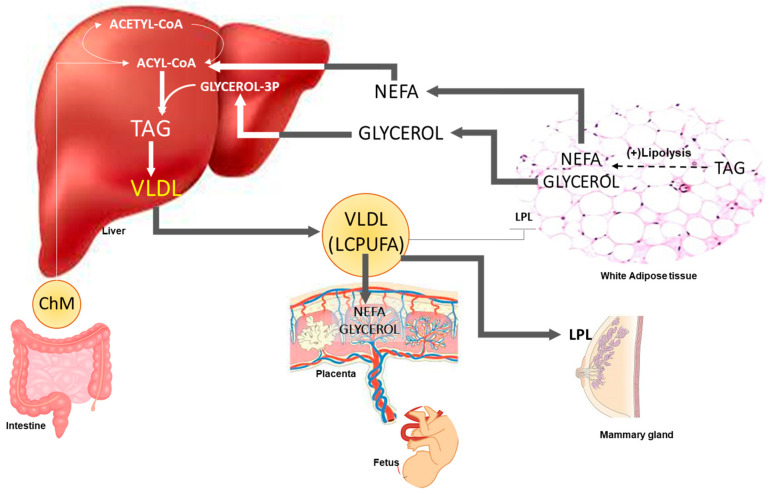
Schematic view of main lipid metabolic adaptations taking place during late pregnancy. A decline in white adipose tissue lipoprotein lipase activity (LPL) occurs, whereas there is an increase in lipolytic activity. The products, in the form of non-esterified fatty acids (NEFAs), including long-chain polyunsaturated fatty acids (LCPUFAs) accumulated at earlier stages of gestation and glycerol, mainly travel to the liver where, among other metabolic fates, they are used for the synthesis of triacylglycerols (TAGs), which are released to the circulation as components of very-low-density lipoproteins (VLDL), a process which is very active in late pregnancy. Plasma VLDL and chylomicrons (ChM) derived from intestinal gut absorption arrive at the placenta, which has the appropriate biochemical machinery to take up these lipoproteins and release the LCPUFAs that are carried into the fetal circulation. Also, close to parturition, an induction of LPL occurs in mammary glands, causing a considerable uptake of TAGs from the lipoproteins in the maternal circulation by this organ, which uses the LCPUFAs it takes up for milk synthesis.

Maternal insulin in early pregnancy must play an important role in the increased size of fat depots characteristic of normal pregnancy during the first two thirds of gestation. The secretion of insulin is increased in early pregnancy before changes in insulin sensitivity occur [78] and the responsiveness of adipose tissue to insulin has been shown to be increased in rats during early pregnancy [79]. From early pregnancy, maternal hyperphagia is normally present, which must also contribute to the increased adipose tissue mass. These changes take place before the decline of post-heparin lipoprotein lipase (LPL) that occurs during late pregnancy in humans [80], indicating that during early pregnancy, the adipose tissue uptake of circulating triacylglycerols (TAGs) would be facilitated, including dietary-derived LCPUFAs, which would be temporarily stored, allowing their release during late pregnancy to become available to the fetus. In fact, it is known that the composition of dietary fatty acids determines their composition in human adipose tissue [81]. During late pregnancy, fat uptake by adipose tissue is decreased as result of a decline in LPL activity [80,82], whereas lipolytic activity in adipose tissue is increased [83], all of which causes a net breakdown of fat depots, including the release of stored LCPUFAs in the form of non-esterified fatty acids (NEFAs) that, as reported in pregnant rats, are transferred to the maternal liver where they are re-esterified to form triacylglycerols that are released back to the circulation as components of very-low-density lipoproteins (VLDL). That is why most LCPUFAs in maternal circulation are in their esterified form associated to plasma lipoproteins rather than in their NEFAs form [84,85], and this is how they reach the placenta to be released into fetal circulation after further lipolysis as NEFAs [86]. A schematic view of these main lipid metabolic adaptations taking place during late pregnancy are shown in Figure 4. 

At postpartum, maternal hypertriacylglycerolemia rapidly ceases [80], and the effect may be associated with the rapid increase in LPL activity in mammary glands and its low activity in adipose tissue, as found in rats [87,88], which facilitate an increased uptake of circulating triacylglycerols by mammary glands instead of their uptake by adipose tissue. In fact, these changes facilitate the uptake of diet-derived LCPUFAs by the mammary gland and their presence in the milk [89,90]. 

In whole animal trials of pigs, we studied whether diet-derived LCPUFAs during early pregnancy were stored in maternal adipose tissue to be released to maternal circulation at late pregnancy, crossing the placenta to become available to the fetus, and around parturition to be taken up by maternal mammary glands, becoming available to the suckling newborn [38]. A fish-oil-supplemented diet was given during the first half of gestation to pregnant sows and were compared to those fed an olive-oil-supplemented diet. The fish oil diet produced an increase in maternal plasma DHA levels at the end of pregnancy and were also increased in the colostrum and milk during lactation as well as in the plasma of suckling piglets. These findings show that during the first half of pregnancy, maternal adipose tissue plays an important role in the storage of dietary LCPUFAs, which are then mobilized around parturition and early lactation for milk synthesis [38]. 

Based on all the preceding findings, we propose the following scheme: during early pregnancy, dietary LCPUFAs are stored in maternal adipose tissue. However, during late pregnancy, an enhanced adipose tissue lipolytic activity causes those LCPUFAs to be released into the circulation to become available to the maternal liver, where they become components of VLDL that return to the circulation. After being taken up by the placenta, those lipoproteins become an important source of LCPUFAs for the fetus. Moreover, around parturition, those LCPUFAs together with those of dietary origin are diverted to the mammary gland and are used for milk synthesis to become available to the newborn during suckling.

## 5. Dietary Fatty Acid during Pregnancy and Lactation

### 5.1. Sources of Maternal LCPUFAs to Sustain Fetal Development

The supply of EFAs and LCPUFAs from the diet becomes important during pregnancy, in particular during the third trimester of gestation, for the optimal development of brain and retina. As mentioned above, DHA and AA are of special importance for fetal development. These LCPUFAs can be synthesized by the fetus from the EFAs as early as 26 weeks of gestation [91], but since the fetus has only limited desaturase activity [92], the amount of LCPUFA synthesis from EFAs seems to be negligible in the human fetus [93], which therefore is very dependent on their placental transfer from maternal circulation. Most of the fat deposition in the human fetus takes place in the last 10 weeks of pregnancy [94]. However, the metabolic elongation and desaturation of LA and ALA to form AA and DHA, occur effectively during the first days of life in humans, including very immature preterm neonates [91], and breastfed newborns synthesize LCPUFAs already during the first week of life [92]. 

A low intake of EFAs during gestation is associated with a reduced neonatal growth, and although the percentage of certain LCPUFAs, like AA and DHA, in healthy maternal plasma correlates with those in the fetus or newborn [95], this is not the case when those fatty acids (i.e., AA and DHA) are expressed as absolute concentrations [96]. In fact, although it was initially reported that the percentage of AA and DHA is higher in cord blood serum than in maternal serum, and the term “biomagnification” was coined for this phenomenon [97], it was later found that their actual concentrations were lower in the cord serum than in mothers [96], and so, the use of the term is not justified.

In both n-3 and n-6 polyunsaturated families, many forms of PUFAs exists. As mentioned above in Section 2.1 and Figure 2, n-3 and n-6 PUFAs are competitively metabolized by the same set of desaturation, elongation and oxygenase enzymes, although Δ6D appears to have a greater affinity for ALA than for LA [98], which are the main substrates of the enzyme. Despite this, the conversion of ALA into LCPUFAs is sensitive to dietary LA content. Thus, the excess of certain dietary fatty acids like the excessive intake of vegetable oils rich in LA like safflower, sunflower or corn oils may inhibit the Δ6D and reduce the synthesis of DHA from ALA (18:3 n-3). Also, the synthesis of AA may be decreased by an excess of LA [99]. Moreover, desaturases and elongases are also regulated by LCPUFA-produced desaturation pathways, since both animal [12] and human [100] studies coincide in observing that when fish oil (rich in both EPA and DHA) is consumed, low plasma levels of AA are found.

In any case, pregnancy is associated with an increased need for LCPUFAs, as well as its possible depletion in maternal stores [101]. Considering the importance of DHA and AA for brain and retinal functioning during fetal development, there are studies which have analyzed the effects of supplementing the maternal diet with LCPUFAs [102]. Preterm infants have lower DHA and AA statuses than full-term neonates [58], probably as a consequence of not receiving the supply of EFAs and LCPUFAs during their third trimester, and it has been found that supplementation with DHA in the neonatal period improves both visual acuity and brain functions in preterm infants [103,104].

It is true that dietary supplementation with fish oils rich in n-3 LCPUFAs during pregnancy has produced confusing results. There are studies showing that an increased intake of seafood and n-3 LCPUFA supplements prevent preterm birth, promoting longer gestation [105]; however, there are also studies that have found no such association [106,107]. Intervention trials, which provided fish oil supplements during the last trimester of pregnancy, failed to show any added benefit over supplementation with soy oil on the development or behavior of infants [108]. The effects of DHA supplementation (400 mg/day) from mid-gestation until birth in a low-income area showed no differences versus controls in birth weight, birth length or head circumference [109]. Recently, it has been found that a high dose of DHA (800 mg/day in pregnant women enrolled before 20 weeks) reduced the rate of both early preterm birth (<34 weeks) and preterm birth (<37 weeks of gestation) [110]. Other recent studies provide results along the same line [111,112]. Moreover, as pointed out by Hofmeyr et al. [113], based on a large antenatal care framework by the WHO, “if further research substantiates the preventive effect of three additional interventions (supplementation with omega-3 fatty acids, calcium and zinc) on small vulnerable newborns, millions of low birth weights births and neonatal mortality could be avoided per year”.

Although there are conflicting results on the efficacy of DHA supplementation, it reduces the effects of prenatal stress and the perinatal mortality rate and increases the birth weight.

### 5.2. Dietary Fatty Acid Supplements during Pregnancy and Lactation in Fetus

In the fetus and newborn, the capacity for the elongation and desaturation of EFAs is insufficient, and consequently, maternal LCPUFA status during pregnancy and lactation becomes critical to allow for the appropriate availability of LCPUFAs to the fetus and newborn infants [95,114,115]. Despite several controlled trials having been carried out to determine whether dietary LCPUFA supplements during different perinatal stages can improve the development outcome, no definitive conclusions have been reached [7,116,117], but some specific comments deserve mention. 

In an intervention trial study in a low-income area involving fish oil supplements (4 g/day) versus a soy oil control group during the last trimester of pregnancy, no effect on psychomotor development or infant behavior was found [108]. A similar negative finding at birth was found in another intervention study with DHA (400 mg/day) versus placebo from mid-pregnancy until term in another low-income area [109]. In a better nourished area, women receiving DHA-rich fish oil capsules (800 mg/day) versus those receiving vegetable oil capsules without DHA from less than 21 weeks of pregnancy until delivery did not show any significant benefit of DHA in cognitive and language development in their offspring at 18 months of age [118]. However, these results could be due to multiple factors that may favor an absence of effect from the fatty acid supplementation, such as sources of DHA (food vs. supplement; plant vs. marine), type of DHA (mixture vs. purified), trial duration, population characteristics, genetic variation, among others. 

Human breast milk contains DHA and AA [119], with levels that are quite variable across different populations [120]. Moreover, the content of DHA in infant formula is also variable [121] and a question is raised concerning the presence or absence of both AA and DHA [122]. In any case, it has been proven that breastfeeding has a slightly better neurological development in children at different ages than formula feeding [123,124,125]. However, it is not clear whether the composition of the breast milk is responsible for the different effects because the prolongation of the breastfeeding alone produces a better cognitive development [126]. The supplementation of formula with LCPUFAs has been associated with beneficial effects on neurodevelopmental outcomes of full-term infants until the age of four months [127], but when outcomes of the LCPUFA-supplemented formula were measured between 6 and 24 months of age, no effects were found [128,129], indicating no long-term beneficial effects. In another study, a biphasic response between breastfeeding and formula has been described, showing a better neurological status at three months of age in breastfed infants than in those fed with formula, whereas no difference was found at 18 months [130,131], but the difference re-emerged at 42 months of age [132]. Further studies are needed to confirm this biphasic response. 

### 5.3. Dietary Fatty Acid Supplements during Pregnancy and Lactation in Preterm Newborns

Preterm infants are particularly vulnerable to a deficiency of LCPUFAs, and are most likely to benefit from LCPUFA supplementation. 

Classical lipid emulsions currently available for the maintenance of nutritional delivery after birth were 100% soybean oil-based lipid emulsion, with a high content of the essential fatty acids LA and ALA [133]. However, an analysis of circulating fatty acid levels in postnatal period for infants receiving these lipid emulsions early in life revealed increasing circulating amounts of those fatty acids but continued deficits of AA and DHA [134], as predicted by the low capacity of preterm infants to transform these precursors into the downstream fatty acids AA and DHA adequately [135]. In contrast to soy-based emulsions, those based on fish oil (either 100% or the newer emulsions based on mixtures with other oils), provide EPA and DHA to increase postnatal circulating levels of these LCPUFAs, but also increase the levels of LA less dramatically than soybean oil emulsions [136]. However, fish-oil-containing emulsions could produce a decrease in circulating levels of AA [136,137] as well as in the n-6/n-3 ratio in preterm infants, when compared with those found in breastfed neonates born at term to mothers consuming western diets. In fact, it has been suggested that rather than the type of lipid emulsion used, the decrease in AA may be behind the adverse effects associated with these formulations [135], such as an increased risk of premature retinopathy [138] or late-onset sepsis [139]. Currently, there is insufficient evidence to establish whether one of the currently available emulsions is any better than the others. This is because of the heterogeneity of many clinical trials designed to evaluate the beneficial effects of supplementation with LCPUFAs for preterm infants, with low subject numbers, late or inadequate dosing of LCPUFAs, or the inclusion of heterogeneous populations of preterm infants—very immature, critically ill preterm, among others. 

Further research is required to establish the ideal composition of lipid emulsion for preterm infants. Correcting the fatty acid balance will be a major goal of those studies, but will also need to address the role of the downstream mediators of LCPUFAs in postnatal development. AA and DHA can be oxidized via enzymatic processes or via non-enzymatic free radical oxidation to produce a variety of biologically active molecules known as oxylipins [140,141]. These include the pro-inflammatory leukotrienes and anti-inflammatory lipoxins derived from AA [142], and the resolvins and protectins derived from EPA and DHA [142,143], with anti-inflammatory effects [144]. In animal models, it has been shown that supplementation with n-3 PUFAs resulted in a reduction in levels of pro-inflammatory oxylipins derived from AA [145]. Interestingly, recent studies indicate that parenteral lipid emulsion fortified in n-3 PUFAs generates an anti-inflammatory profile by reducing the levels of AA-derived oxylipins and increasing the levels of the pro-resolving ALA-related and DHA-related oxylipins [146]; this may be of benefit given that inflammation is felt to contribute to the morbidities of the preterm infant. However, nowadays, there is little information regarding the oxylipid profile generated by parenteral lipid emulsions in preterm infants, and larger multicenter cohort studies are necessary to further elucidate the role of oxylipins in neonatal development and disease.

In addition, another aspect to consider when optimizing the formulation of lipid emulsions is the fact that, regardless of the type of lipid emulsion used, fatty acids are found esterified as TAGs, phospholipids or cholesteryl esters. Therefore, the tolerance of lipid emulsions is based on the ability to hydrolyze serum TAGs into NEFAs, which depends on LPL activity. However, in preterm infants, LPL activity is low, as seen in small for gestational age infants [147], who have low LPL levels, little adipose tissue and low tissue levels of carnitine. Therefore, the inability to clear TAGs effectively could be associated with lipid intolerance and an increase in the probability of complications involved with the administration of lipid infusions [148]. The fact that recent studies have shown that dietary DHA from TAGs or from natural phosphatidylcholine (*sn*-2 position) is not suitable for brain enrichment, whereas DHA from lysophosphatidylcholine efficiently enriches the brain and is functionally effective [149], invites us to investigate how fatty acid carriers in dietary lipid emulsions can be improved.

The ESPGHAN/ESPEN/ESPR/CSPEN has summarized the guidelines of pediatric parenteral lipids administration [150]. It is pointed out that intravenous lipid emulsions are an indispensable part of pediatric parenteral nutrition, providing essential fatty acids and helping with the delivery of lipid-soluble vitamins, and the use of the type of lipid emulsions are critically analyzed.

## 6. Summary

Certain limitations exist for mammals to modify fatty acid changes. Besides the role of arachidonic acid and other LCPUFAs for the synthesis of eicosanoids, different LCPUFAs have many other effects, including their abilities to regulate gene expression and downstream events. LCPUFAs are susceptible to autoxidation, which is prevented by the action of antioxidants in the form of certain enzymes and compounds that protect against oxidation or repair the damage caused. Under normal conditions, the fetus needs both EFAs and LCPUFAs, which are obtained from its mother by placental transfer. In early pregnancy, dietary-derived fatty acids are accumulated in maternal adipose tissue. However, during late pregnancy, corresponding to the period of the highest fetal growth, maternal adipose tissue becomes catabolic and LCPUFAs are released into the circulation by adipose lipolytic activity. The released LCPUFAs are taken up by maternal liver to be esterified and released back to the circulation as TAGs in VLDL that become available to the placenta to be transferred to the fetus in the form of NEFAs. An enhanced adipose tissue lipolysis is maintained around parturition and esterified LCPUFAs are diverted to mammary glands thanks to an increased activity of lipoprotein lipase for milk production. Throughout this process, LCPUFAs become available to the newborn during suckling. The important role of both DHA and AA for the development of the nervous system and for growth has motivated their dietary supplement during different postnatal stages. This has been especially important in preterm infants both because under normal conditions, the fetus acquires most of these fatty acids during late pregnancy, and because the immaturity of the enzyme systems for the synthesis of AA and DHA from their respective EFAs.

Essential fatty acids and their LCPUFAs derivatives play a major role during pregnancy and the perinatal period. They provide the precursor for eicosanoids and they are important constituents of all cell membranes. But beyond these functions, there is increasing evidence to indicate the association of these fatty acids with a reduction in the occurrence of obstetrics complications, and the improvement of perinatal outcomes such as preterm delivery and intrauterine growth retardation. Therefore, fatty acids like LA, AA and DHA may be of great importance in the diet of pregnant women and may also influence offspring metabolic health. However, more studies are necessary to identify specifically targeted metabolic vulnerable populations, that would benefit most from an increase in EFAs and LCPUFAs in the diet of pregnant women. Moreover, beyond the competitive interaction that omega-6 and omega-3 LCPUFAs exert on each other, it is necessary to determine if there is a potential competition of DHA intake on EPA and AA metabolism, in order to make better dietary recommendations during pregnancy and perinatal states.

## Data Availability

Not applicable.
